# Supplementary material for: Splice-Junction-Based Mapping of Alternative Isoforms in the Human Proteome
Source: Cell Rep. Author manuscript; Available in PMC 2020 Jan 15. (PMC6961840; doi:10.1016/j.celrep.2019.11.026)
Supplement: 2 [file NIHMS1546469-supplement-2.zip › DF1/PXD010154/Lung/Lung_33_PPP1R12A_ISEMEEELKR.pdf]

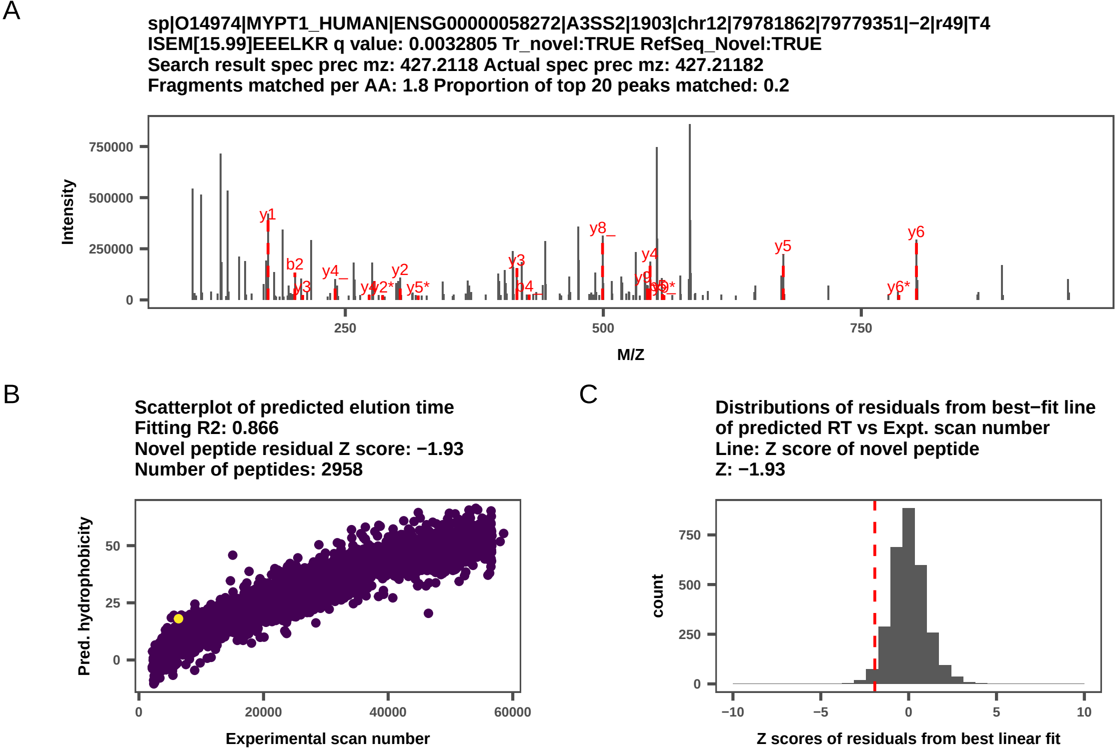
A
sp|O14974|MYPT1_HUMAN|ENSG00000058272|A3SS2|1903|chr12|79781862|79779351|−2|r49|T4
ISEM[15.99]EEELKR q value: 0.0032805 Tr_novel:TRUE RefSeq_Novel:TRUE
Search result spec prec mz: 427.2118 Actual spec prec mz: 427.21182
Fragments matched per AA: 1.8 Proportion of top 20 peaks matched: 0.2
Intensity
750000
500000
250000
0
y1
b2
y3
y4_
y2
y5*
y3
b4
y8_
y4
y5
y6
y6*
250
500
750
M/Z
B
Scatterplot of predicted elution time
Fitting R2: 0.866
Novel peptide residual Z score: −1.93
Number of peptides: 2958
Pred. hydrophobicity
50
25
0
0
20000
40000
60000
Experimental scan number
C
Distributions of residuals from best−fit line
of predicted RT vs Expt. scan number
Line: Z score of novel peptide
Z: −1.93
count
750
500
250
0
−10
−5
0
5
10
Z scores of residuals from best linear fit
